# Metastatic gastric adenocarcinoma with appendiceal phlegmon: a case report

**DOI:** 10.1186/s13256-023-03787-3

**Published:** 2023-02-27

**Authors:** Farshid Mohammadi, Mehta Razzaghi, Sajad Mousivand, Ehsan Amjadinia

**Affiliations:** 1 Clinical Research Development Unit of Shahid Beheshti Hospital, Hamadan University of Medical Science, Hamadan, Iran; 2 Clinical Research Development Unit of Besat Hospital, Hamadan University of Medical Science, Hamadan, Iran

**Keywords:** Metastatic gastric cancer, Appendicitis, Phlegmon, Case report

## Abstract

**Background:**

Gastric carcinoma is one of the most frequent malignancies worldwide. Gastric cancer metastasis to the appendix is uncommon in incidence, and has been rarely described in acute-appendicitis-related literature reviews. In this presented case, we have reported a rare case of appendiceal phlegmon, due to a diagnosis of metastatic gastric adenocarcinoma with uncommon symptoms.

**Case presentation:**

A 79-year-old Caucasian male presented to the emergency department with history of weakness, anorexia, lethargy, and mood changes for 2 months. Abdominopelvic computed tomography showed an abscess in right iliac muscle. After percutaneous drainage of the abscess and 6 weeks antibiotic therapy, an appendectomy was done for the patient. Histopathologic findings revealed the involvement of the appendiceal wall by adenocarcinoma, most probably with gastrointestinal origin. Gastric cancer was confirmed later by upper endoscopy and pathologic report.

**Conclusions:**

Although the presence of tumor in appendectomy specimens is rare, and metastasis to appendix is even rarer, it should be considered as a cause of appendicitis, especially in elderly patients.

## Background

Gastric carcinoma is one of the most prevalent malignancies around the world [1]. Gastric adenocarcinoma, with a prevalence of 85% among gastric cancers, is considered the third most common cause of cancer mortality worldwide [2]. Most patients with gastric carcinoma are symptomatic, and are often diagnosed in advanced stages of the disease when they have metastatic lesions in other organs [3]. Gastric cancer disseminates through straight development across the gastric wall to the perigastric organs (liver and pancreas) and lymphatics, and results in seeding of peritoneal regions [2]. Liver, peritoneum, lung, and bone are the most frequent sites of gastric metastasis. The average survival after diagnosis in metastatic gastric carcinoma is 3 months, worse among individuals with metastasis to bone and liver (2 months) [4].

Gastric cancer metastasis to the appendix is uncommon in terms of incidence, and has been rarely described in acute-appendicitis-related literature reviews [5–8]. In this presented case, we have reported a case of appendiceal phlegmon, due to the diagnosis of metastatic gastric adenocarcinoma admitted with septic shock.

## Case presentation

### Patient information

A 79-year-old Persian male presented to the emergency department with history of weakness, anorexia, drowsiness, and mood changes for 2 months. He was a shopkeeper, and before the onset of symptoms, he regularly performed activities such as cycling and walking, but in the last 2 months he had lost his ability, with his symptoms having worsened in the last 2 weeks. There was no complaint of fever, nausea and vomiting, headache, abdominal pain, altered bowel habits, and other gastrointestinal (GI) symptoms. His past medical history included hypertension, ischemic heart disease, previous coronary artery bypass surgery, and an ischemic cerebral vascular accident episode 3 months prior to his admittance. He had no positive family history of diseases, including cancer, vascular, or heart problems.

### Clinical and laboratory findings

Physical examination showed a low-grade fever [temperature (T), 37.9 °C], hypotension [blood pressure (BP) 83/55 mmHg], pulse rate (PR) of 88 beats per minute, and respiratory rate (RR) of 16 breaths per minute. There was no sign of tenderness, or rebound tenderness, on abdominal examination, and the only positive finding was a bilateral basal reduction of respiratory sounds on lung auscultation.

He was admitted to the emergency department with an impression of “sepsis,” based on a positive Quick Sepsis Related Organ Failure Assessment (qSOFA) score.

Initial laboratory tests revealed leukocytosis [white blood cell count (WBC), 14,700 with 80% neutrophils], normochromic normocytic anemia [hemoglobin (Hb), 11.9 g/dL; mean corpuscular volume (MCV), 86; mean corpuscular hemoglobin (MCH), 30 pg], and high erythrocyte sedimentation rate (ESR, 103 mm/hour) and C-reactive protein (CRP^+++^) levels. The patient was admitted to the intensive care unit (ICU) and treated with broad-spectrum antibiotics. The lung computed tomography revealed moderate bilateral pleural effusion and passive collapse consolidation of the lower lobes of basal segments. Thoracentesis demonstrated transudative effusion, and echocardiography showed heart failure with reduced ejection fraction (HFrEF-EF, 40%).

#### Treatment and management

**Fig. 1:**
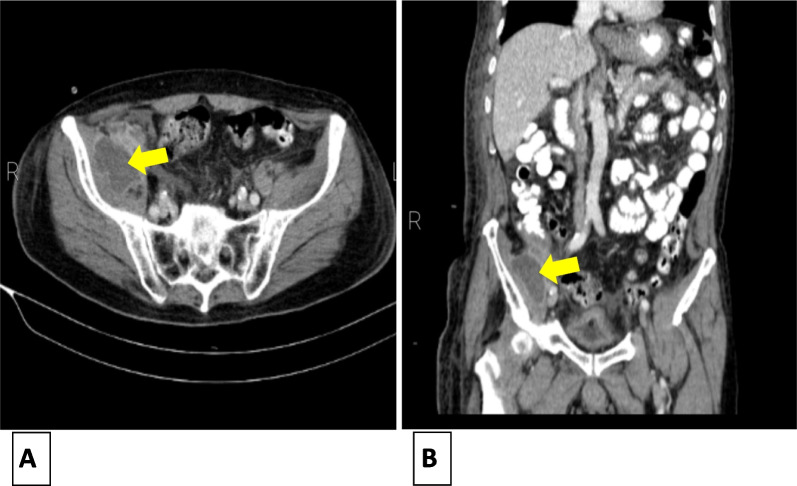
Abdominopelvic CT scan of the patient. Yellow arrows demonstrate collection in the right iliac muscle adjacent to the appendix. **A** Axial view. **B** Coronal view

There were no positive findings on abdominopelvic ultrasonography. Since the source of infection was not found, an abdominopelvic computed tomography examination was performed, which showed 35 × 52 mm abscess formation in the right iliac muscle and dilated appendix with peripheral inflammation (Fig. 1).

**Fig. 2:**
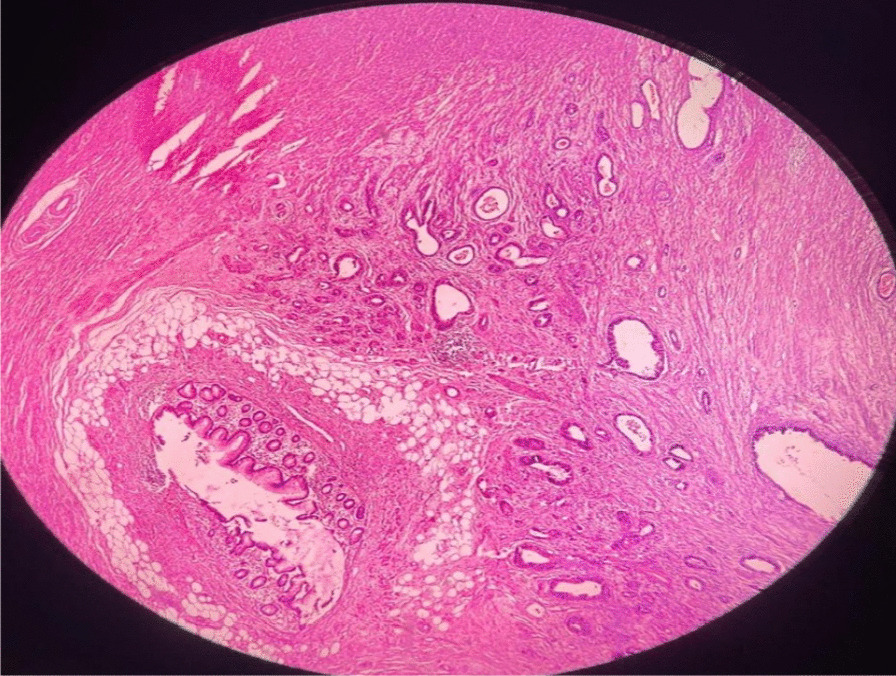
Histopathology findings reveal infiltration of some atypical epithelial cells, with pleomorphic and hyperchromic nuclei, within the muscularis propria layer, which are arranged as glandular structures

On the basis of these findings, percutaneous drainage was done under a guided ultrasound, and antibiotic therapy was continued. After these interventions, the patient’s hypotension and mood changes improved. Subsequent tests revealed improved leukocytosis (WBC, 5100 cell/microliter) and anemia (Hb, 12.5 gr/dL); however, the ESR level was still high (87 mm/hour). Due to the iliac abscess and changes in the appendix location, the patient underwent antibiotic therapy for 6 weeks, with suspected appendiceal phlegmon and subsequently, an interval appendectomy was performed after 6 weeks. The pathology report showed involvement of the appendiceal wall by tumoral glands (adenocarcinoma), most probably of gastrointestinal origin. Consequently, a colonoscopy was performed to determine the origin of the tumor, and there were no positive pathological findings except a few polyps in the transverse colon. Eventually, the patient underwent upper endoscopy, which revealed an infiltrative and circumferential tumoral stenosis in the body of the stomach. The pathology report showed severe dysplastic gastric mucosa, with an ulcerative feature compatible with gastric well-differentiated adenocarcinoma (Fig. 2). Due to distant metastasis, the patient was referred to the oncology ward with diagnosis of stage IV gastric cancer and underwent chemotherapy. According to age, physical conditions, and clinical judgment of the oncologist, the oral chemotherapy drug capecitabine was started for the patient.

## Discussion

There is limited evidence in studies describing the presence of appendiceal metastasis as the first manifestation of primary cancer. Araujo *et al*. study showed that incidental appendiceal tumor followed by appendectomy occurs in only 0.9% of patients and metastasis to the appendix is very rare and the most origin sites are lungs, stomach and colon [9]. Yoon *et al*. found that appendiceal metastasis is usually associated with an extensive stage of disease, in which the average survival rate was 22.6 months. The combination of the female sex, simultaneous diagnosis of appendiceal metastasis with the primary tumor, and initiation of chemotherapy ensured a longer survival [10].

Gastric carcinoma is one of the most common malignancies around the world and the patients are already in the advanced stages of such incurable disease [1–3]. The average prognosis in metastatic gastric carcinoma is 3 months, which is worse among individuals with metastasis to bone and liver (2 months) [4].

Although gastric cancer metastasis to the appendix is uncommon, it can remain asymptomatic and be diagnosed accidentally [8]. However, in the most documented cases, due to adherence of metastatic cancer cells to the serosa and infiltration of all the appendix layers, lumen obstruction can develop, leading to inflammation, appendicitis symptoms, and perforation [7]. The mechanism of metastasis remains unknown, but it has been considered that the appendix is infiltrated by peritoneal seeding, or even indicates a single peritoneal mass [6].

Gastric adenocarcinoma is radioresistant neoplasm, and for sufficient control of the primary tumor, doses of external beam irradiation are required, specifically which overpass adjacent organs’ resistance, for example, bowel mucosa. Therefore, pain relief is the prominent role of radiation therapy in patients with metastatic gastric carcinoma. Among patients with metastatic gastric adenocarcinoma, 30–50% respond relatively well to a combination of cytotoxic agents. Regardless of the response rates, complete remission is rare, partial responses are temporary, and the general effect of combination therapy on survival is low [2].

Thirteen to twenty percent of acute appendicitis cases lead to perforation, with higher rates occurring in men and elderly adults [11]. In imaging studies, appendicitis may present with a localized perforation (an inflammatory lesion frequently referred to as a “phlegmon”) or, infrequently, a free perforation. The management of perforated appendicitis is determined by the patient’s situation (stable versus unstable), the type of perforation, and whether an abscess or phlegmon is present in imaging studies [12]. Stable patients with extensive (> 3 cm) abscesses must first be managed with intravenous antibiotics and percutaneous drainage [13]. Urgent surgery in patients presenting with symptoms over a long period, and phlegmon or abscess development, has been shown to lead to increased morbidity due to firm adhesion and inflammation. Conservative management of the initial admission enables a decrease in regional inflammation, and interval appendectomy can be performed at a lower risk [14]. Following favorable conservative management of perforated appendicitis, all patients should undergo follow-up in 6–8 weeks, to carry out interval appendectomy to rule out an appendiceal tumor. The appendiceal tumor is much more prevalent in the interval appendectomy specimens (10–29%), compared with conventional appendectomy specimens (0.9–1.4%), particularly in patients over 40 years old [15, 16].

## Conclusion

We have reported a rare case of appendiceal metastasis, with phlegmon, as the first presentation of gastric adenocarcinoma. Although appendicitis caused by metastasis to the appendix is unusual in clinical practice, it should be considered, especially in older patients.

## Data Availability

All patient’s data are available in the hospital’s data archive system and, if needed, can be made available through the corresponding author of the article.
